# Serum biomarker for diagnostic evaluation of pulmonary arterial hypertension in systemic sclerosis

**DOI:** 10.1186/s13075-018-1679-8

**Published:** 2018-08-16

**Authors:** Lisa M. Rice, Julio C. Mantero, Eric A. Stratton, Rod Warburton, Kari Roberts, Nicholas Hill, Robert W. Simms, Robyn Domsic, Harrison W. Farber, Robert Layfatis

**Affiliations:** 1 Boston University School of Medicine, E5 Arthritis Center, 72 E Concord Street, Boston, MA 0211 USA; 2 Tufts University, Boston, MA USA; 3 University of Pittsburgh Medical Center, Pittsburgh, PA USA

**Keywords:** Classification, Proteomic, Scleroderma, Biomarkers, Pulmonary arterial hypertension

## Abstract

**Background:**

Systemic sclerosis-associated pulmonary arterial hypertension (SSc-PAH) is one of the leading causes of death in SSc. Identification of a serum-based proteomic diagnostic biomarker for SSc-PAH would allow for rapid non-invasive screening and could positively impact patient survival. Identification and validation of novel proteins could potentially facilitate the identification of SSc-PAH, and might also point to important protein mediators in pathogenesis.

**Methods:**

Thirteen treatment-naïve SSc-PAH patients had serum collected at time of diagnosis and were used as the discovery cohort for the protein-expression biomarker. Two proteins, Midkine and Follistatin-like 3 (FSTL3) were then validated by enzyme-linked immunosorbent assays. Midkine and FSTL3 were tested in combination to identify SSc-PAH and were validated in two independent cohorts of SSc-PAH (*n* = 23, *n* = 11).

**Results:**

Eighty-two proteins were found to be differentially regulated in SSc-PAH sera. Two proteins (Midkine and FSTL3) were also shown to be elevated in publicly available data and their expression was evaluated in independent cohorts. In the validation cohorts, the combination of Midkine and FSTL3 had an area under the receiver operating characteristic curve (AUC) of 0.85 and 0.92 with respective corresponding measures of sensitivity of 76% and 91%, and specificity measures of 76% and 80%.

**Conclusions:**

These findings indicate that there is a clear delineation between overall protein expression in sera from SSc patients and those with SSc-PAH. The combination of Midkine and FSTL3 can serve as an SSc-PAH biomarker and are potential drug targets for this rare disease population.

**Electronic supplementary material:**

The online version of this article (10.1186/s13075-018-1679-8) contains supplementary material, which is available to authorized users.

## Background

Cardiopulmonary involvement is the most common cause of morbidity and mortality in patients with systemic sclerosis (SSc). Twelve percent of SSc patients will develop SSc-associated pulmonary arterial hypertension (SSc-PAH); these patients have an estimated 50% 3-year survival [1]. Early and accurate diagnosis of PAH is clinically challenging and relies on right heart catheterization (RHC). RHC is invasive, is not suitable for screening, and so is typically performed only on patients with a high index of suspicion based on echocardiogram and other criteria that are neither highly sensitive nor specific [2–4].

Earlier diagnosis and treatment with PAH-specific therapeutics is associated with better outcomes in patients with SSc-PAH [5, 6]. Thus, identification of a diagnostic biomarker that would allow for rapid non-invasive screening could positively impact patient survival, supplement current screening methods, and potentially reduce the need for diagnostic RHC.

Transthoracic echocardiography is the most widely accepted screening tool for the diagnosis of SSc-PAH. Echocardiographically estimated systolic PAP (sPAP) correlates reasonably (*r* = 0.83) with RHC [7], though it has potential limitations including an ~ 45% false positive rate [8–10]. Echocardiogram has also been studied in combination with other measures. PAH biomarkers such as brain natriuretic peptide (BNP) and the N-terminal fragment of pro-BNP (NT-proBNP) have similar diagnostic accuracy as the echocardiogram [11]. Natriuretic peptide levels of have been shown to correlate with hemodynamic measurements, predict disease prognosis and mortality among those with PAH [12]. They are also generally specific for SSc-PAH, but lack sensitivity [13]. As such, other potential biomarkers, such as osteopontin, pentraxin 3, C-reactive protein (CRP), interleukin (IL)1, IL8 and tumor necrosis factor alpha (TNFα) have been investigated as diagnostic tools. In preliminary studies, some have appeared superior to both BNP and NT-proBNP [14–17]. However, these findings are tempered by the fact that they are single studies and have yet to be validated.

The clinical use of gene expression for classifying different biological disease states is well established [18]. We have previously described SSc-PAH associated gene-expression patterns in peripheral blood mononuclear cells (PBMCs) that highlight IFN-regulation and monocyte/macrophage activation in disease pathogenesis [17, 19]. Thus, we hypothesized that wide-scale proteomic studies of serum might identify a diagnostic biomarker of SSc-PAH. This hypothesis was further supported by our previous small proteomic study, in which we identified 17 cytokines associated with SSc-PAH [17].

In the present study, we utilized a proteomic technology to identify novel proteins specific to the diagnosis of SSc-PAH. We show that in combination, Follistatin-like 3 (FSTL3) and Midkine (MDK) are highly diagnostic for SSc-PAH, and have validated these biomarkers in two independent cohorts. These results suggest that serum assays in patients with PAH could be dramatically improved with identification of these serum biomarkers and, thus, aid in the earlier diagnosis of SSc-PAH.

## Methods

### Patient selection

Institutional review boards at Boston University, University of Pittsburgh, and Tufts University approved the collection of all serum samples and clinical data. Written informed consent was obtained from all patients before sample collection. Diagnosis of PAH was made by RHC: mean pulmonary artery pressure (mPAP) of ≥ 25 mmHg, a pulmonary capillary wedge pressure of ≤ 15 mmHg, and a pulmonary vascular resistance of ≥ 3 Wood units [20]. Diagnosis of SSc was made by a rheumatologist and date of disease onset is recorded as date of first non-Raynaud’s symptom. There are two subcategories of SSc, limited cutaneous systemic sclerosis (lcSSc) and diffuse cutaneous systemic sclerosis (dcSSc). This distinction is based on extent and location of skin disease. The majority of patients included in this study were lcSSc patients with limited skin disease, any exceptions are noted in the detailed description of each patient cohort included. All samples in the discovery cohort (*n* = 13) were collected at Boston University. Patients had limited skin disease (lcSSc) and were PAH treatment-naïve. Sera were collected at time of diagnostic RHC and were analyzed by SOMAscan (SomaLogic Boulder, CO, USA), which allowed for the capability to examine 1129 proteins simultaneously. Sixteen subjects with lcSSc and no clinical evidence of PAH (medical records were reviewed by a clinical expert in the field, forced vital capacity [FVC] > 70%, negative RHC, or no record of an abnormal finding on an echocardiograph) or interstitial lung disease (ILD), diagnosed by high-resolution CT scans (HRCTs), served as controls. Protein data were analyzed for differences in lcSSc-PAH patients (Wilcoxon signed-rank test). Analytes for further study were selected based on the q values (false discovery rates [FDR] < 0.1). These parsed analytes were clustered and visualized using the R environment for statistical computing (version 3.2.1). The clustering was unsupervised and used Spearman’s correlation and average linkage. Identification of pathways linked to the differentially expressed proteins was performed using Qiagen’s Ingenuity Pathway Analysis (IPA, Qiagen, Redwood City, CA, USA). Subjects in validation cohorts diagnosed with ILD based on HRCTs were included when measured FVC was > 70%. The discovery cohort was from Boston University, and the validation cohort one was from both Tufts University as well as Boston University and validation cohort two was from University of Pittsburgh.

Subjects enrolled with signed informed written consent by the institutional review board of Boston University also included patients with dcSSc, as well as lcSSc subjects showing evidence of ILD (confirmed by HRCT) and healthy controls. Sera from these subjects were also analyzed by SOMAScan, after collection into a serum separator tube, allowed to clot, aliquoted, and stored at −80 °C.

### ELISA validations and statistical analysis

Concentrations of candidate proteins were measured in duplicate using commercial ELISA kits for FSTL3 (DFLRG0: R&D Systems, Minneapolis, MN, USA), or for MDK (ab193761: Abcam, Cambridge, MA, USA).

Putative biomarker protein concentrations were log2-transformed to better approximate a normal distribution and obtain reliable odds ratio (OR) estimates [21]. Wilcoxon signed-rank test were used to test the difference in SSc analyte concentrations from lcSSc-PAH. Models of discreet variables were developed using logistic regression and multiple logistic regression. Sensitivity and specificity of discrete variables were assessed using receiver operating characteristic (ROC) curves, plotting the sensitivity on the y-axis and 1-specificity on the x-axis. The area under the curve (AUC) of a ROC was computed to provide a global measure of performance and for comparing biomarker performance, with AUC of 0.90–.1 considered excellent, 0.80–0.90 considered good, 0.7–0.8 considered fair, 0.6–0.7 considered poor, 0.5–0.06 considered without value. Youden’s J statistic was used to calculate the best threshold level of the biomarker that gives equal weight to sensitivity and specificity [22].

Publicly available gene expression data were downloaded from GEO: http://www.ncbi.nlm.nih.gov/geo/. The data included five data sets with relevant SSc subpopulations. The first data set was derived from the peripheral blood mononuclear cells (PBMCs) of lcSSc subjects with (*n* = 15) and without (*n* = 21) PAH. The data set was downloaded from GEO accession number GSE19617 [17]. A second data set from the PBMCs of PAH subjects including a systemic sclerosis subset. The data was downloaded from GEO accession number GSE33463 [23]. lcSSc subjects were with (*n* = 37) and without (*n* = 19) PAH. The third data set was derived from PBMCs of patients with SSc-PAH (*n* = 10) and SSc without (n = 10). The data set was downloaded with the GEO accession number GSE22356 [24]. The second type of data was derived from lung tissue from transplant subjects, there were *n* = 6 Ssc subjects with PAH and *n* = 9 subjects with normal lungs. The data set was downloaded with the GEO accession number GSE48149 [25]. The third type of data was derived from skin tissue of SSc subjects (*n* = 61) and healthy controls (*n* = 36). The data set was downloaded with the GEO accession number GSE58095 [26].

## Results

### Pathway analysis of protein expression

**Fig. 1:**
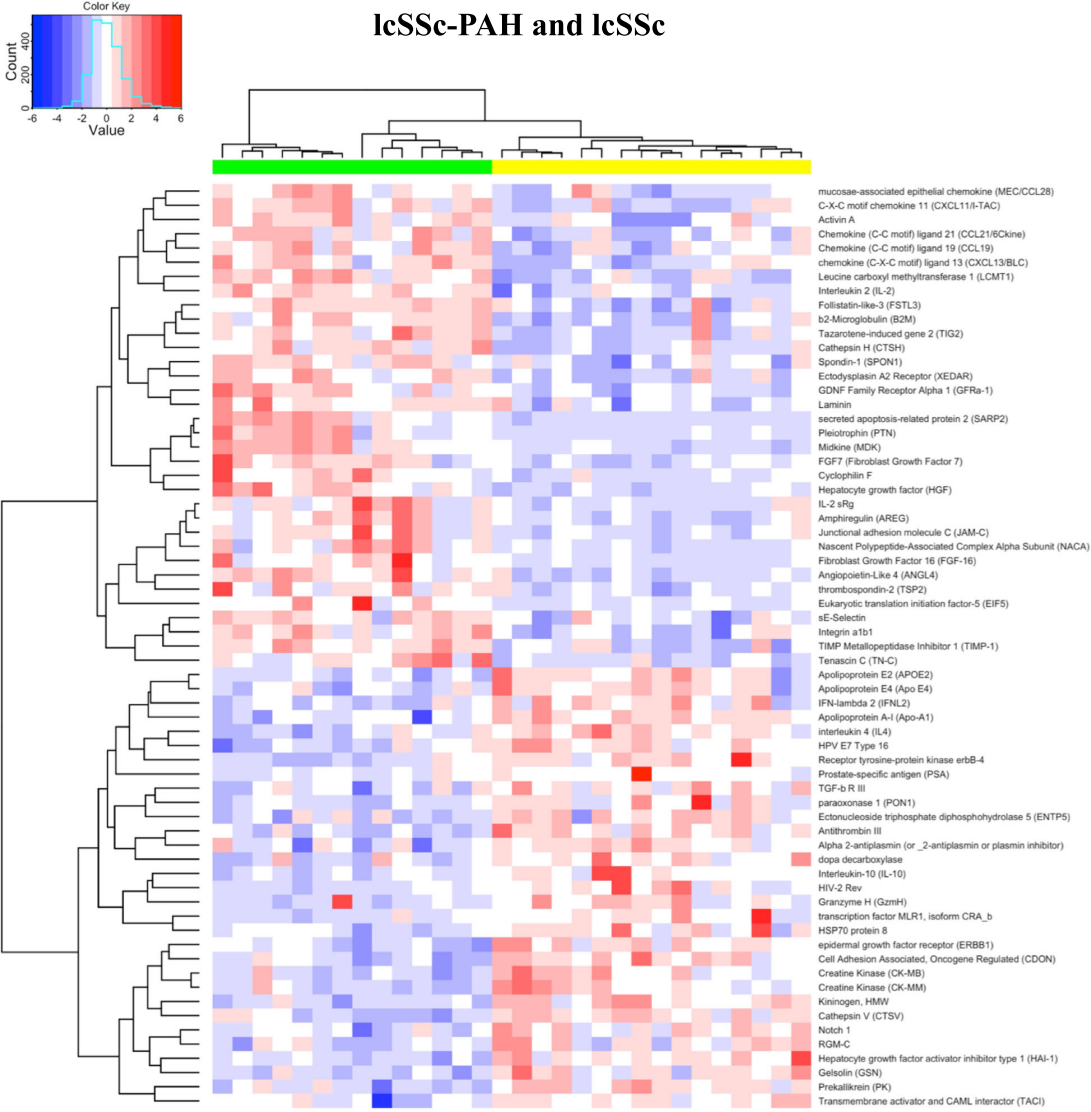
Differential regulation of sera protein expression in lcSSc-PAH patients. Unsupervised hierarchal clustering of lcSSc-PAH patients (*green color bar*) and lcSSc patients with no lung disease (*yellow color bar*). Here, *red* and *blue* indicate high or low expression

SOMAScan protein expression was evaluated in sera from 13 subjects with lcSSc-PAH and 16 subjects with lcSSc and no clinical evidence of PAH (Table 1). Detailed hemodynamic data are found in Additional file 1: Table S1. Eighty-two proteins were found to be differentially regulated in lcSSc-PAH patients compared to lcSSc controls (FDR q ≤ 0.1, summarized in Additional file 2: Table S2. Of the 82 proteins that were significantly different between comparing subjects with lcSSc-PAH to lcSSc-no PAH 32 were increased and 50 decreased. Analytes meeting these criteria were analyzed by unsupervised clustering for both proteins and subjects. lcSSc-PAH and lcSSc controls clustered independently (Fig. 1). An unfiltered clustering diagram of all the measured proteins was also examined. The presence of PAH was the dominant signal in the serum proteome, as all the lcSSc-PAH cases clustered together without any structured stratification of groups (Additional file 3: Figure S1).

**Table 1:** Clinical characteristics

Baseline demographics	Discovery cohort		Validation cohort 1		Validation cohort 2	
	lcSSc-PAH	lcSSc-no PAH	lcSSc-PAH	lcSSc-no PAH	lcSSc-PAH	lcSSc-no PAH
Age (year)	*n* = 13	*n* = 16	*n* = 23	*n* = 12	*n* = 11	*n* = 18
Mean (SD)	65 (7.4)	50 (14)	66 (8.6)	54 (17.8)	66 (9.4)	62 (8.5)
Median (range)	65 (56–81)	57 (25–70)	64 (52–85)	52 (26–76)	66 (52–81)	63 (46–79)
Sex						
Female, % (n)	92% (12)	87.5% (14)	83% (19)	92% (11)	100% (11)	83% (15)
Male,% (n)	8% (1)	12.5% (2)	17% (4)	8% (1)	0% (0)	17% (3)
mPAP (mmHg)						
Mean (SD)	46 (9.1)	–	42 (12.3)	–	40 (10.8)	–
Median (range)	45 (34–68)	–	43 (26–69)	–	42 (25–54)	–
mPCWP (mmHg)						
Mean (SD)	10 (2.7)	–	10 (4.3)	–	10 (3.2)	–
Median (range)	11 (4–14)	–	11 (1–15)	–	9 (5–15)	–
PVR (Woods units)						
Mean (SD)	9 (4.9)	–	8 (5.3)	–	8 (4.1)^a^	–
Median (range)	8 (5–24)	–	6 (3–27)	–	7 (3–13)^a^	–
ILD (Dx by HRCT)						
Positive, % (n)	7% (1)	–	13% (3)	–	0% (0)	–
Negative, % (n)	83% (12)	–	87% (20)	–	100% (11)	–
Treatment						
Treated, % (n)	0% (0)	–	48% (11)	≈	100% (11)	–
Untreated, % (n)	100% (13)	–	52% (12)	–	0% (0)	–
BNP (pg/mL)						
Mean (SD)	261 (313)	–	411 (494)	–	–	–
Median (range)	140 (42–1054)	–	148 (12–1630)	–	–	–
Date of sample						
At time of RHC, % (n)	100% (13)	–	91% (21)	–	36% (4)	–

^a^Missing values

BNP, brain natriuretic peptide; lcSSc-PAH, limited cutaneous systemic sclerosis pulmonary arterial hypertension; ILD, interstitial lung disease; mPAP, mean pulmonary artery pressure; mPCWP, mean pulmonary capillary wedge pressure; PVR, pulmonary vascular resistance; RHC, right heart catheterization

We mined publicly available data to identify which proteins of the 82 had been previously observed as differentially regulated in skin and lung tissue or PBMCs of SSc-PAH patients (Additional file 4: Table S3). The search yielded 16 proteins with corresponding differential expression by either fold change, absolute value log2 fold-change > 1 or adjusted *p* value ≤ 0.1 (*p* value was adjusted by Benjamini-Hochberg). About half of the identified proteins were related to the extracellular matrix including SFRP1, TNC, and TIMP1. Some immune markers were also seen as differentially regulated across data sets including complement decay-accelerating factor (CD55), CD36, CD27, and CCL19 [17, 24]. MDK was the only protein previously observed to also be differentially regulated at the mRNA level in lung tissue [25].

**Fig. 2:**
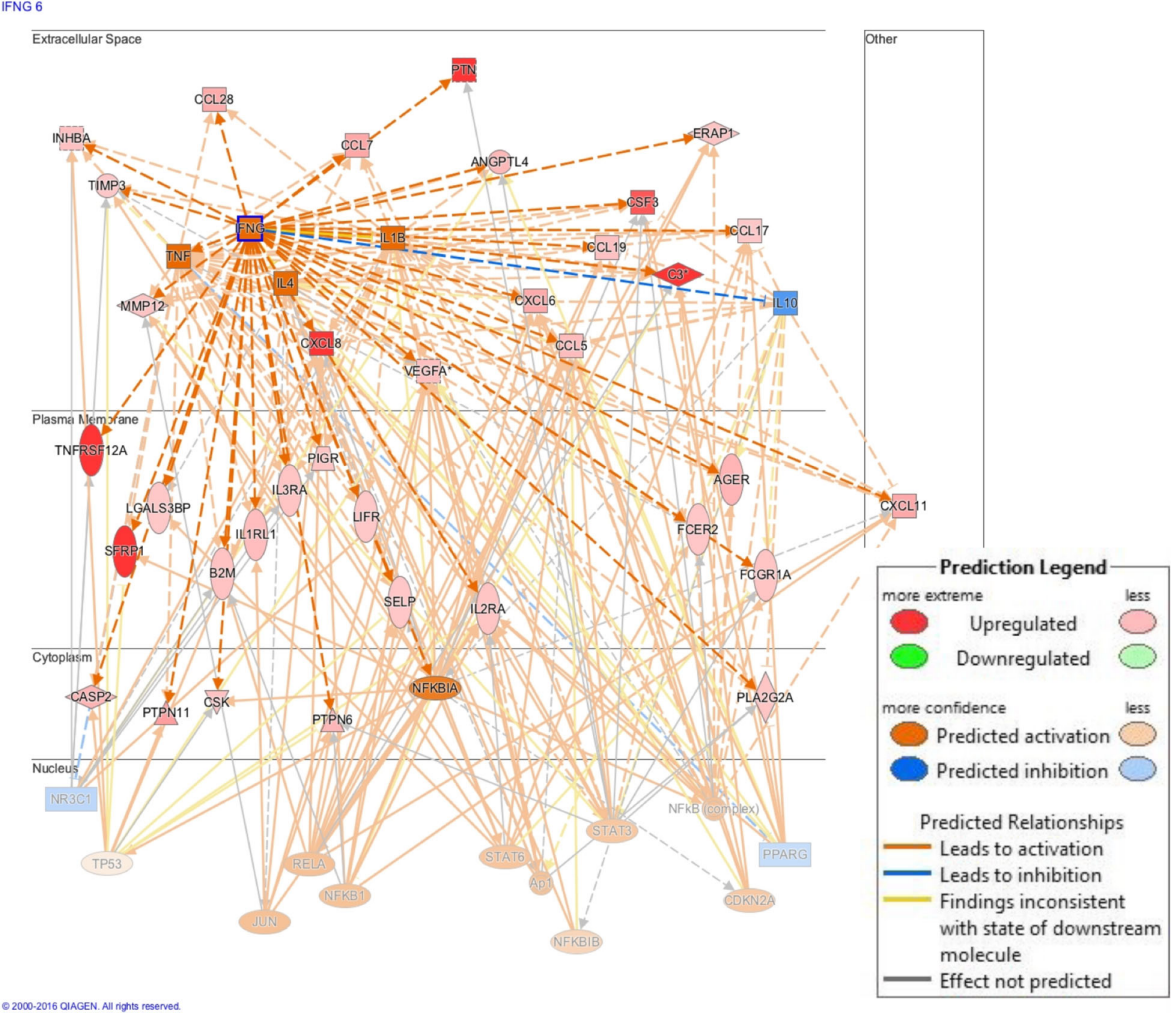
INF-γ pathway signature in sera of lcSSc-PAH patients. Graphical representation of the proteins involved the activated INF-γ signature pathway of lcSSc-PAH patients. Colors indicate predicted activation (*orange*: activated, and *blue*: inhibited) and regulation status (*red* upregulated, *green* downregulated) of molecules in the data set. Lines between molecules depict predicted relationships based on what is known in the current literature

The 82 proteins that were differentially regulated between lcSSc-PAH and lcSSc-no PAH controls were explored in greater depth by pathway analysis. There was a cluster of chemokine signaling molecules activated including CCL19, CXCL13, CXCL11, CCL28, and CCL21, as well as several transforming growth factor beta (TGF-β) regulated molecules including FSTL3 and Spondin-1. Using IPA several activated upstream regulators were identified within the data, including a strong interferon gamma (IFN-γ) signature (Fig. 2).

### Putative biomarker selection and validation

**Fig. 3:**
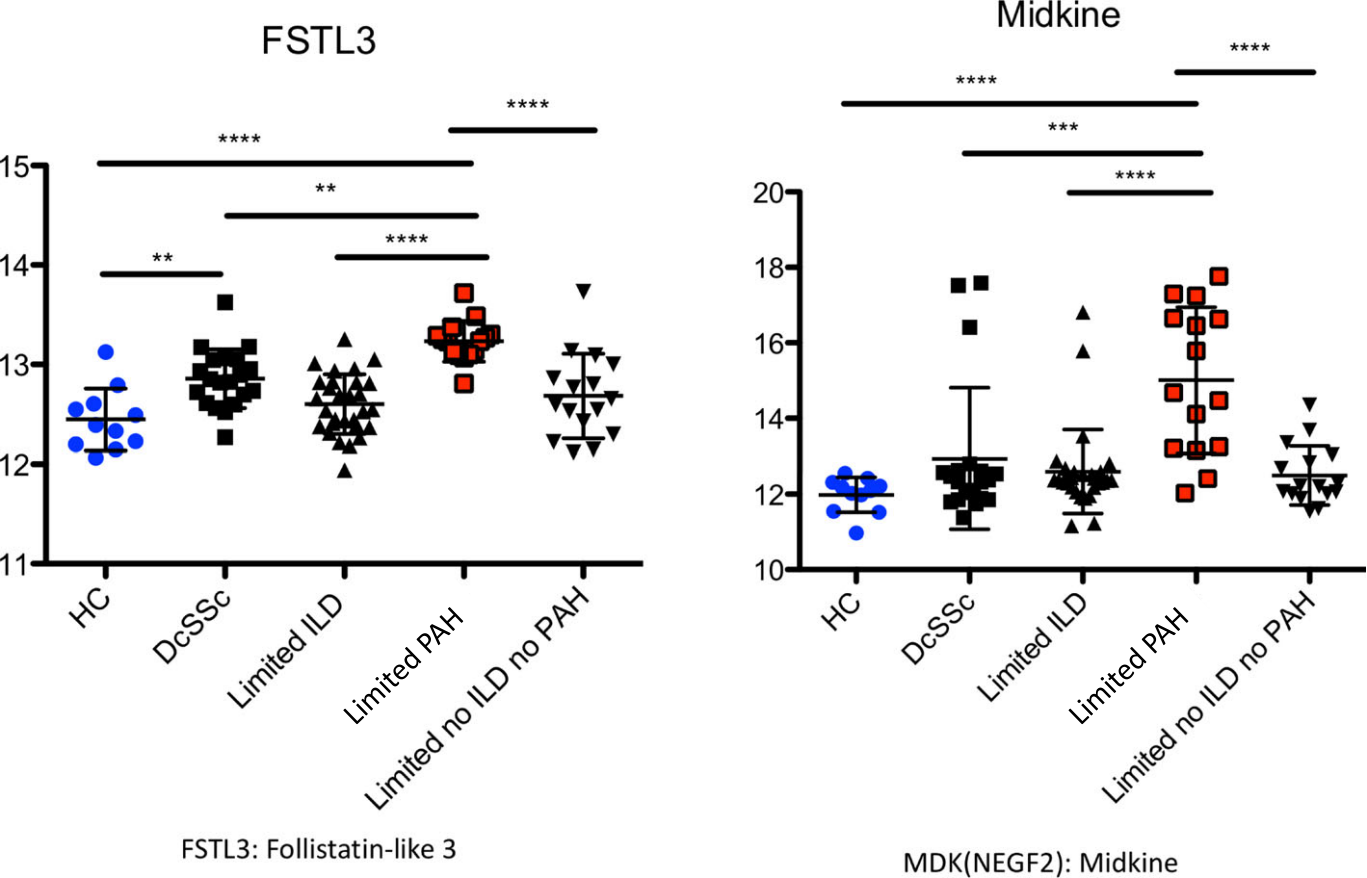
Midkine (MDK) and follistatin-like 3 (FSTL3) are upregulated specifically in the lcSSc-PAH population. Graphs show the differential expression of MDK and FSTL3 relative to healthy controls with a side-by-side comparison to patients with dcSSc, SSc-ILD. Data is displayed as log2 expression levels and comparisons made by ANOVA: corrected with Bonferroni’s multiple comparison test. *dcSsc* diffuse systemic sclerosis *HC* healthy control, *ILD* interstitial lung disease, *lcSSc*-*PAH* limited cutaneous systemic sclerosis pulmonary arterial hypertension

Five of the proteins upregulated in lcSSc-PAH sera (FSTL3, Spondin-1, junctional adhesion molecule C (JAM-C), CCL28, and MDK) were investigated further for their individual ability to discriminate between subjects with lcSSc-PAH and lcSSc-no PAH in silico (logistic regression) based on biological relevance, their presence in different clusters and average fold change > 2 (Additional file 5: Figure S2). FSTL3 and MDK had nearly equal areas under the curve (AUC: 0.90 and 0.89). However, FSTL3 performed better then MDK when high sensitivity was required, and MDK performed better then FSTL3 when high specificity was required. Additionally, MDK and FSTL3 did not correlate with each other or with BNP (Additional file 6: Figure S3). This suggested that in combination these proteins would give additional information to the currently measured BNP. These proteins were investigated in combination using a generalized linear model. MDK and FSTL3 were also examined in other clinical subgroups of systemic sclerosis and were shown to be specifically upregulated compared to dcSSc, lcSSc-ILD (diagnosed by HRCT) (Fig. 3), healthy controls were included for reference.

The ability to discriminate between lcSSc subjects with and without PAH using Midkine and FSTL3 was confirmed by enzyme-linked immunosorbent assay (ELISA) on the patients originally analyzed by SOMAscan. Both proteins were elevated in subjects with SSc-PAH compared to those without PAH, and when modeled together using multiple logistic regression showed an increase in discriminatory power (Additional file 7: Figure S4).

**Fig. 4:**
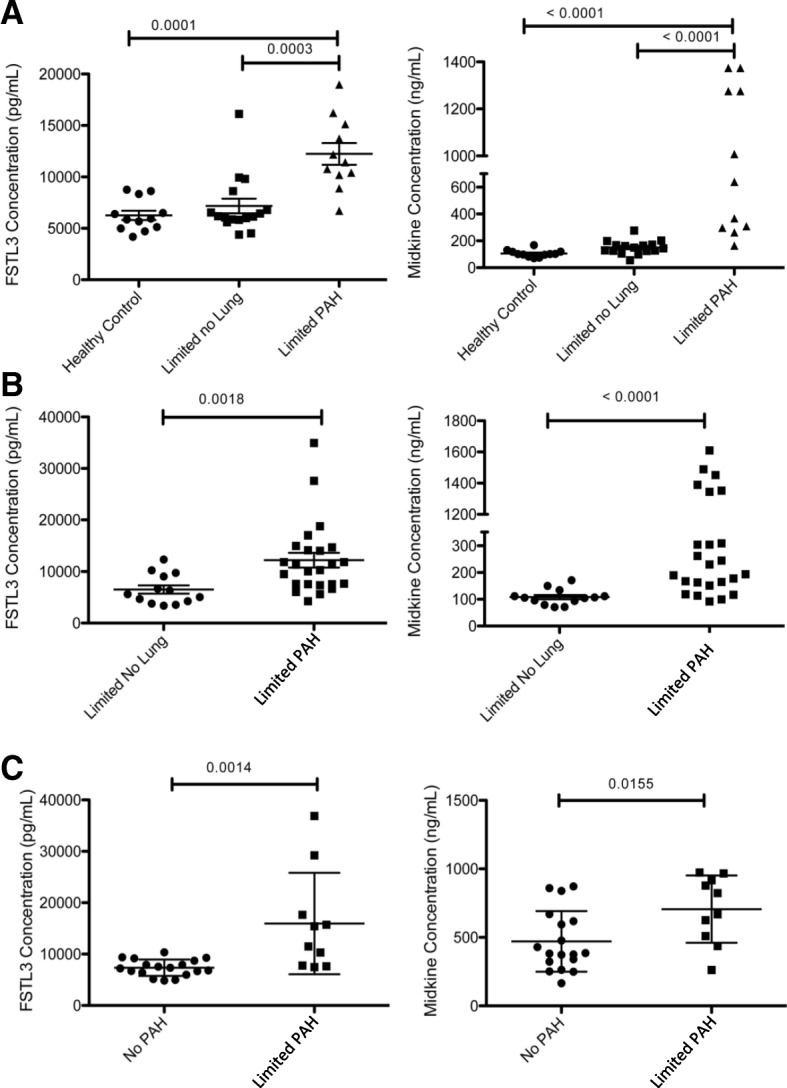
ELISA validation cohort data. Graphs show differences (Wilcoxon signed-rank test) of lcSSc-PAH Follistatin-like 3 (FSTL3), and Midkine (MDK) protein concentrations as compared to SSc patients with no lung disease in the test cohort (A) as well as two independent validation cohorts (B and C). *PAH* pulmonary arterial hypertension

**Fig. 5:**
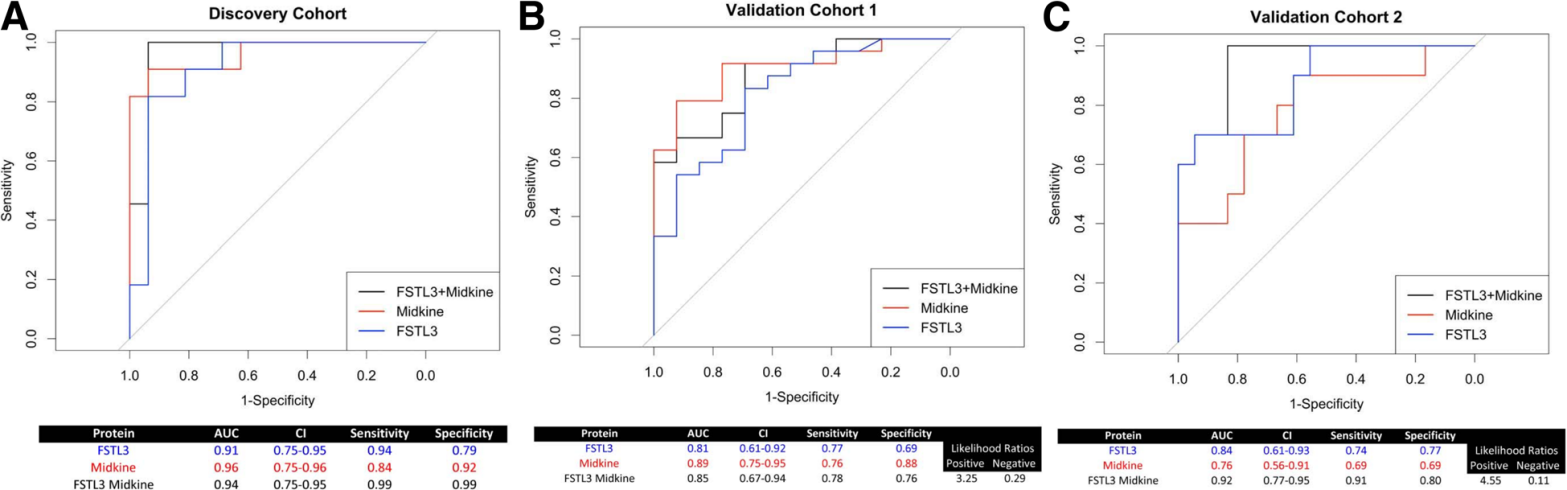
Receiver operator characteristic curves of ELISA data. Graphs show differences Receiver operator characteristic curves of lcSSc-PAH follistatin-like 3 (FSTL3), and Midkine (MDK) protein concentrations as well as the two in combination as compared to lcSSc patients with no lung disease in the test cohort (A) as well as two independent validation cohorts (B and C)

Sera from two validation cohorts were then used to test the discrimination of Midkine and FSTL3 for lcSSc-PAH. Clinical characteristics in the validation cohorts were similar but not identical to the test cohort (Table 1, Additional file 1: Table S1 and Additional file 7: Table S4). In the validation cohorts not all samples were collected on the day of diagnostic RHC; as such, some patients were being treated for PAH. Additionally, some patients had other pulmonary complications, such as ILD. Yet, both cohorts demonstrated a consistent and significant increase (*p* < 0.01) in both FSTL3 and MDK protein concentrations in SSc-PAH patients compared to lcSSc-no PAH (Fig. 4). Logistic and multiple logistic models were created based on protein concentrations (Fig. 5). In the discovery cohort, the biomarker had an area under the ROC curve (AUC) of 0.94 (95% confidence interval (CI) 81 to 95), a sensitivity of 99%, and a specificity of 99%. In the first validation cohort, the biomarker had an AUC of 85% (95% CI 67 to 94), a sensitivity of 76%, and a specificity of 76%. In the second validation cohort, the biomarker had an AUC of 92% (95% CI 77 to 95), a sensitivity of 91%, and a specificity of 80%.

The discovery cohort was comprised of all untreated patients and one subject with ILD, while the second validation cohort was comprised of all treated patients with no subjects with ILD (Table 1, Additional file 1: Table S1, and Additional file 8: Table S4). However, the first validation cohort had a mix of treated (*n* = 11) and untreated (*n* = 12) subjects as well as a mix of subjects with (*n* = 3) and without (*n* = 20) ILD (Table 1, Additional file 8: Table S4). After excluding subjects with ILD and patients treated for PAH, FSTL3 and MDK remained elevated (*p* < 0.05) in SSc-PAH patients compared to lcSSc-no PAH (Additional file 9: Figure S5). However, exclusion of these patients did not increase the parameters of the ROCs, with the AUC including all the patients remaining the highest at 85% (Additional file 9: Figure S5).

## Discussion

Investigation into the circulating proteome of lcSSc-PAH allowed for the identification of multiple putative biomarkers. Our results show a circulating serum protein pattern that is associated with lcSSc-PAH compared to lcSSc-no PAH, thus identifying a potential serum proteomic biomarker for use in the diagnostic evaluation of lcSSc-PAH. In sum, we demonstrated that the combination of FSTL3 and MDK are highly associated with lcSSc-PAH.

Our results show that 82 circulating serum proteins are associated with lcSSc-PAH compared to lcSSc-no PAH; this data set allowed us a unique opportunity to examine potential upstream regulators of these proteins. Within those 82 differentially regulated proteins, there were a number of activated chemokines, CCL19, CXCL13, and CCL21, which are implicated in tertiary lymphoid structures [27]. In addition, many of the upregulated proteins are regulated by INF-γ, thus confirming previously published gene-expression patterns that observed INF regulation in PBMCs of SSc-PAH [19].

From these 82 differentially regulated proteins, we showed that the combination of FSTL3 and MDK are highly associated for SSc-PAH in the independent cohorts, with an average sensitivity of 84% and an average specificity of 79%. These findings are plausible pathophysiologically. FSTL3, a TGF-β regulated protein, inhibits the protective actions of activin A on cardiac myocytes. We also previously observed that FSTL3 is elevated, compared to healthy controls, in the sera, PBMCs, and skin of patients with dcSSc [28]. The growth factor, MDK, has been implicated in the pathogenesis of hypertension, kidney disease, and lung fibrosis [29–31].

The lack of effect of various treatments in the validation data sets on biomarker performance suggests that this is a robust diagnostic biomarker. Also, that the roles of these biomarkers are upstream from the pathophysiologic processes affected by vasodilators, the most commonly available therapeutics. Additionally, some patients had radiographic evidence of ILD as well as SSc-PAH. The effect of ILD and PAH treatment was tested in a small subset of patients in validation cohort one. Including patients with ILD also bestowed no additional benefit to the diagnostic capabilities of FSTL3 and MDK.

Some samples were not collected at the time of diagnostic RHC (Table 1 and Additional file 9: Table S4) and thus the relationship between biomarker and clinical disease is less certain in these cases. In spite of this, there was still a significant increase in MDK and FSTL3 among patients with SSc-PAH. Of particular interest are those patients whose sera was collected prior to PAH diagnosis suggesting that these biomarkers might be elevated before PAH is clinically suspected. There has not been investigation of MDK or FSTL3 in other subset of SSc patients who might have increased concentrations of those specific proteins, such as patients with cardiac involvement or renal disease.

## Conclusions

In sum, we have identified serum proteins that are strongly associated with SSc-PAH. Further investigation of these and other proteins has the potential to improve the diagnosis, care, and outcomes in this clinically challenging PAH subset.

## Additional files

Additional file 1: **Table S1.** Detailed hemodynamic measurements and clinical characteristics of the discovery cohort. (PDF 127 kb)

Additional file 2: **Table S2.** Complete list of differentially regulated proteins: 82 proteins were found to be differentially regulated in lcSSc-PAH patients compared to lcSSc controls (false discovery rate q ≤ 0.1). (PDF 1235 kb)

Additional file 3: **Figure S1.** Unfiltered clustering diagram of all 1129 measured proteins: Unsupervised hierarchal clustering of lcSSc-PAH patients (*green color bar*) and lcSSc patients with no lung disease (*yellow color bar*). Here, *red* and *blue* indicate high or low expression. (PDF 1774 kb)

Additional file 4: **Table S3.** Shortlist proteins previously observed as differentially regulated in skin and lung tissue or PBMCs of SSc-PAH patients. (PDF 71 kb)

Additional file 5: **Figure S2.** Receiver operator characteristic (ROC) curves of SOMAlogic: ROCs based on in silico (logistic regression). (PDF 678 kb)

Additional file 6: **Figure S3.** Correlations of Midkine (MDK), follistatin-like 3 (FSTL3), and BNP: protein levels of MDK, FSTL3, and BNP are plotted as linear regressions Spearman’s correlation coefficients are reported in the *upper left corner* of each graphic. (A) Examines the correlation between FSTL3 and MDK, (B) the correlation between FSTL3 and BNP, (C) MDK and BNP. (PDF 361 kb)

Additional file 7: **Figure S4.** Concentrations of Midkine (MDK), follistatin-like 3 (FSTL3): concentrations of FSTL3 (A) and Midkine (B) were measured by ELISA and modeled together using multiple logistic regression (C). (PDF 779 kb)

Additional file 8: **Table S4.** Detailed hemodynamic measurements and clinical characteristics of validation cohorts: Table A contains the details from validation cohort one while Table B contains the details from validation cohort two. (PDF 334 kb)

Additional file 9: **Figure S5.** Concentrations of Midkine (MDK), follistatin-like 3 (FSTL3): concentrations of FSTL3 and MDK were examined with treated subjects excluded from the analysis (A) or subjects with ILD (B). ROC parameters were examined with the exclusion of the different subsets (C). (PDF 970 kb)

## Abbreviations

AUC: Area under the receiver operating characteristic curve
BNP: Brain natriuretic peptide
dcSSc: Diffuse systemic sclerosis
FDR: False discovery rate
FSTL3: Follistatin-like 3
FVC: Forced vital capacity
HRCT: High-resolution CT scan
ILD: Interstitial lung disease
INF-γ: Interferon gamma
lcSSc: Limited cutaneous systemic sclerosis
MDK: Midkine
mPAP: Mean pulmonary artery pressure
NT-proBNP: N-terminal fragment of pro-BNP
PBMC: Peripheral blood mononuclear cell
RHC: Right heart catheterization
ROC: Receiver operating characteristic curve
sPAP: Systolic PAP
SSc: Systemic sclerosis
SSc-PAH: Systemic sclerosis-associated pulmonary arterial hypertension
TNF-β: Transforming growth factor beta
