# Correction: Serum biomarker for diagnostic evaluation of pulmonary arterial hypertension in systemic sclerosis

**DOI:** 10.1186/s13075-022-02816-8

**Published:** 2022-05-23

**Authors:** Lisa M. Rice, Julio C. Mantero, Eric A. Stratton, Rod Warburton, Kari Roberts, Nicholas Hill, Robert W. Simms, Robyn Domsic, Harrison W. Farber, Robert Lafyatis

**Affiliations:** 1 Boston University School of Medicine, E5 Arthritis Center, 72 E Concord Street, Boston, MA 0211 USA; 2 Tufts University, Boston, MA USA; 3 University of Pittsburgh Medical Center, Pittsburgh, PA USA

**Correction: Arthritis Res Ther 20, 185 (2018)**

**https://doi.org/10.1186/s13075-018-1679-8**

Following publication of the original article [1], the authors identified an error in the author name of Robert Lafyatis.

The incorrect author name is: Robert Layfatis

The correct author name is: Robert Lafyatis

The author group has been updated above.

## References

[1] Rice LM, Mantero JC, Stratton EA (2018). Serum biomarker for diagnostic evaluation of pulmonary arterial hypertension in systemic sclerosis. Arthritis Res Ther.

