# Supplementary material for: Serum biomarker for diagnostic evaluation of pulmonary arterial hypertension in systemic sclerosis
Source: Arthritis Res Ther. 2018 Aug 16;20:185. doi: 10.1186/s13075-018-1679-8 (PMC6097341; doi:10.1186/s13075-018-1679-8)
Supplement: Supplementary file 1 — Table S1. Detailed hemodynamic measurements and clinical characteristics of the discovery cohort. (PDF 127 kb) [file 13075_2018_1679_MOESM1_ESM.pdf]

Additional Table 1: Detailed hemodynamic measurements and clinical characteristics of discovery cohort

							Hemodynamic Measurements								
**StudyID**	**Sex**	**Age**	**WHO class**	**PAH**	**Treated**	**Sample at RHC**	**mPCWP (mmHg)**	**mPAP (mmHg)**	**PVR (Woods Units)**	**BNP w/in 1 yr of RHC**	**BNP (pg/mL)**	**ILD**	**HRCT**	**Echo w/in 1 yr of RHC**	**PASP (mmHg)**
**1079**	F	66	III	YES	No	YES	12	60	11.5	YES	61	NO		NO	-
**1140**	F	58	III	YES	No	YES	9	43	5.5	YES	178	NO	YES	YES	80
**1164**	F	70	II	YES	No	YES	9	48	8.8	YES	71	NO		NO	-
**1293**	F	57	II	YES	No	YES	11	43	7.9	YES	154	YES	YES	NO	-
**1314**	F	59	III	YES	No	YES*	13	68	23.6	No	49	NO	YES	YES	90
**1315**	F	68	II	YES	No	YES	7	45	8.0	YES	100	NO		NO	-
**1335**	F	59	II	YES	No	YES	4	40	13.3	-	-	NO	YES	NO	-
**1369**	F	65	-	YES	No	YES	8	34	4.7	-	-	NO		YES	-
**1372**	F	70	-	YES	No	YES	14	47	9.2	YES	498	NO	YES	YES	64
**1375**	F	56	II	YES	No	YES	9	46	6.4	YES	42	NO		NO	-
**1382**	F	81	-	YES	No	YES	11	49	8.8	YES	1054	NO		YES	87
**1388**	F	64	-	YES	No	YES	11	37	6.5	YES	140	NO		YES	106
**1420**	M	74	II	YES	No	YES	12	42	7.4	YES	524	NO		YES	84

*blood taken within a week
