# Supplementary material for: Serum biomarker for diagnostic evaluation of pulmonary arterial hypertension in systemic sclerosis
Source: Arthritis Res Ther. 2018 Aug 16;20:185. doi: 10.1186/s13075-018-1679-8 (PMC6097341; doi:10.1186/s13075-018-1679-8)
Supplement: Supplementary file 2 — Table S2. Complete list of differentially regulated proteins: 82 proteins were found to be differentially regulated in lcSSc-PAH patients compared to lcSSc controls (false discovery rate q ≤ 0.1). (PDF 1235 kb) [file 13075_2018_1679_MOESM2_ESM.pdf]

Names	TargetFullName	EntrezGeneSymbol	P. Value	Adjusted P	Log FC
Midkine	Midkine	MDK	0.01	0.15	2.54
SARP-2	Secreted frizzled-related protein 1	SFRP1	0.01	0.14	2.50
I-TAC	C-X-C motif chemokine 11	CXCL11	0.00	0.08	1.02
sRAGE	Advanced glycosylation end product-specific receptor, soluble	AGER	0.01	0.14	0.92
BLC	C-X-C motif chemokine 13	CXCL13	0.00	0.05	0.89
NACA	Nascent polypeptide-associated complex subunit alpha	NACA	0.00	0.09	0.80
sE-Selectin	E-Selectin	SELE	0.00	0.09	0.70
6Ckine	C-C motif chemokine 21	CCL21	0.00	0.04	0.70
HGF	Hepatocyte growth factor	HGF	0.00	0.07	0.66
Tenascin	Tenascin	TNC	0.01	0.15	0.65
Ferritin	Ferritin	FTH1 FTL	0.01	0.10	0.65
GFRa-1	GDNF family receptor alpha-1	GFRA1	0.00	0.08	0.64
ANGL4	Angiopoietin-related protein 4	ANGPTL4	0.00	0.08	0.64
AREG	Amphiregulin	AREG	0.01	0.15	0.61
Spondin-1	Spondin-1	SPON1	0.00	0.06	0.55
FSTL3	Follistatin-related protein 3	FSTL3	0.00	0.04	0.55
TIG2	Retinoic acid receptor responder protein 2	RARRES2	0.00	0.03	0.54
Activin A	Inhibin beta A chain	INHBA	0.00	0.05	0.53
b2-Microglobulin	Beta-2-microglobulin	B2M	0.00	0.07	0.53
FGF7	Fibroblast growth factor 7	FGF7	0.00	0.08	0.51
MIP-3b	C-C motif chemokine 19	CCL19	0.00	0.08	0.50
Integrin a1b1	Integrin alpha-I: beta-1 complex	ITGA1 ITGB1	0.00	0.05	0.49
sFRP-3	Secreted frizzled-related protein 3	FRZB	0.01	0.15	0.42
TIMP-1	Metalloproteinase inhibitor 1	TIMP1	0.00	0.04	0.41
Coagulation Factor IXab	Coagulation factor IXab	F9	0.01	0.15	0.38
Laminin	Laminin	LAMA1 LAMB1 LAMC1	0.00	0.07	0.36
IL-2	Interleukin-2	IL2	0.00	0.01	0.35
XEDAR	Tumor necrosis factor receptor superfamily member 27	EDA2R	0.00	0.04	0.34
IL-2 sRg	Cytokine receptor common subunit gamma	IL2RG	0.01	0.15	0.33
Cathepsin H	Cathepsin H	CTSH	0.00	0.03	0.29
LCMT1	Leucine carboxyl methyltransferase 1	LCMT1	0.00	0.00	0.27
Layilin	Layilin	LAYN	0.01	0.15	0.24
CK-MM	Creatine kinase M-type	CKM	0.01	0.11	-1.20
Apo A-I	Apolipoprotein A-I	APOA1	0.00	0.00	-1.23
Cathepsin V	Cathepsin L2	CTSV	0.00	0.01	-1.46
CDON	Cell adhesion molecule-related/down-regulated by oncogenes	CDON	0.00	0.01	-1.77
HAI-1	Kunitz-type protease inhibitor 1	SPINT1	0.00	0.04	-1.85
Antithrombin III	Antithrombin-III	SERPINC1	0.00	0.03	-2.03
CD36 ANTIGEN	Platelet glycoprotein 4	CD36	0.01	0.14	-2.04
Apo E	Apolipoprotein E	APOE	0.00	0.08	-2.07
Apo E2	Apolipoprotein E (isoform E2)	APOE	0.00	0.06	-2.07
Kininogen, HMW	Kininogen-1	KNG1	0.00	0.01	-2.18
ERBB1	Epidermal growth factor receptor	EGFR	0.00	0.01	-2.23
RGM-C	Hemojuvelin	HFE2	0.00	0.04	-2.25
Prekallikrein	Plasma kallikrein	KLKB1	0.00	0.02	-2.29
Apo E4	Apolipoprotein E (isoform E4)	APOE	0.00	0.07	-2.46
EMR2	EGF-like module-containing mucin-like hormone receptor-like 2	EMR2	0.00	0.08	-2.53
ENTP5	Ectonucleoside triphosphate diphosphohydrolase 5	ENTPD5	0.00	0.03	-2.66
GDF-11	Growth/differentiation factor 11	GDF11	0.00	0.06	-2.69
HSP70 protein 8	Heat shock cognate 71 kDa protein	HSPA8	0.01	0.15	-2.69
DAF	Complement decay-accelerating factor	CD55	0.00	0.07	-2.69
TrkB	BDNF/NT-3 growth factors receptor	NTRK2	0.00	0.04	-2.70
Apo E3	Apolipoprotein E (isoform E3)	APOE	0.01	0.13	-2.72
TGF-b R III	Transforming growth factor beta receptor type 3	TGFBR3	0.00	0.09	-2.73
contactin-1	Contactin-1	CNTN1	0.00	0.07	-2.83
ATS13	A disintegrin and metalloproteinase with thrombospondin motifs 13	ADAMTS13	0.01	0.13	-2.85
SET	Protein SET	SET	0.00	0.05	-2.86
Gelsolin	Gelsolin	GSN	0.00	0.04	-2.91
kallikrein 8	Kallikrein-8	KLK8	0.00	0.10	-2.92
Coagulation Factor XI	Coagulation Factor XI	F11	0.00	0.08	-2.99
Met	Hepatocyte growth factor receptor	MET	0.01	0.12	-3.20
Kallistatin	Kallistatin	SERPINA4	0.00	0.09	-3.29
Afamin	Afamin	AFM	0.01	0.13	-3.32
Galectin-3	Galectin-3	LGALS3	0.01	0.15	-3.48
Notch 1	Neurogenic locus notch homolog protein 1	NOTCH1	0.00	0.04	-3.57
Calcineurin	Calcineurin	PPP3CA PPP3R1	0.01	0.13	-3.57
paraoxonase 1	Serum paraoxonase/arylesterase 1	PON1	0.01	0.15	-3.76
dopa decarboxylase	Aromatic-L-amino-acid decarboxylase	DDC	0.01	0.15	-3.85
a2-Antiplasmin	Alpha-2-antiplasmin	SERPINF2	0.01	0.14	-4.07
ERBB4	Receptor tyrosine-protein kinase erbB-4	ERBB4	0.00	0.07	-4.29
CD27	CD27 antigen	CD27	0.01	0.15	-4.43
KIRR3	Kin of IRRE-like protein 3	KIRREL3	0.00	0.04	-4.60
HIV-2 Rev	Protein Rev_HV2BE	Human-virus	0.01	0.13	-4.67
TACI	Tumor necrosis factor receptor superfamily member 13B	TNFRSF13B	0.00	0.06	-4.70
LRIG3	Leucine-rich repeats and immunoglobulin-like domains protein 3	LRIG3	0.01	0.13	-4.73
Thyroglobulin	Thyroglobulin	TG	0.01	0.13	-5.66
HPV E7 Type 16	Protein E7_HPV16	Human-virus	0.00	0.04	-6.25
IFN-lambda 2	Interferon lambda-2	IFNL2	0.00	0.07	-6.47
IL-20 Ra	Interleukin-20 receptor subunit alpha	IL20RA	0.01	0.13	-6.90
IL-4	Interleukin-4	IL4	0.00	0.02	-7.29
ADAMTS-4	A disintegrin and metalloproteinase with thrombospondin motifs 4	ADAMTS4	0.00	0.08	-7.52
GIIE	Group IIE secretory phospholipase A2	PLA2G2E	0.01	0.15	-8.48

Additional Table 2: Complete list of differentially regulated proteins
82-proteins were found to be differentially regulated in lcSSc-PAH patients compared to lcSSc controls (False Discovery Rate q≤0.1)
