# Supplementary material for: Serum biomarker for diagnostic evaluation of pulmonary arterial hypertension in systemic sclerosis
Source: Arthritis Res Ther. 2018 Aug 16;20:185. doi: 10.1186/s13075-018-1679-8 (PMC6097341; doi:10.1186/s13075-018-1679-8)
Supplement: Supplementary file 3 — Figure S1. Unfiltered clustering diagram of all 1129 measured proteins: Unsupervised hierarchal clustering of lcSSc-PAH patients (green color bar) and lcSSc patients with no lung disease (yellow color bar). Here, red and blue indicate high or low expression. (PDF 1774 kb) [file 13075_2018_1679_MOESM3_ESM.pdf]

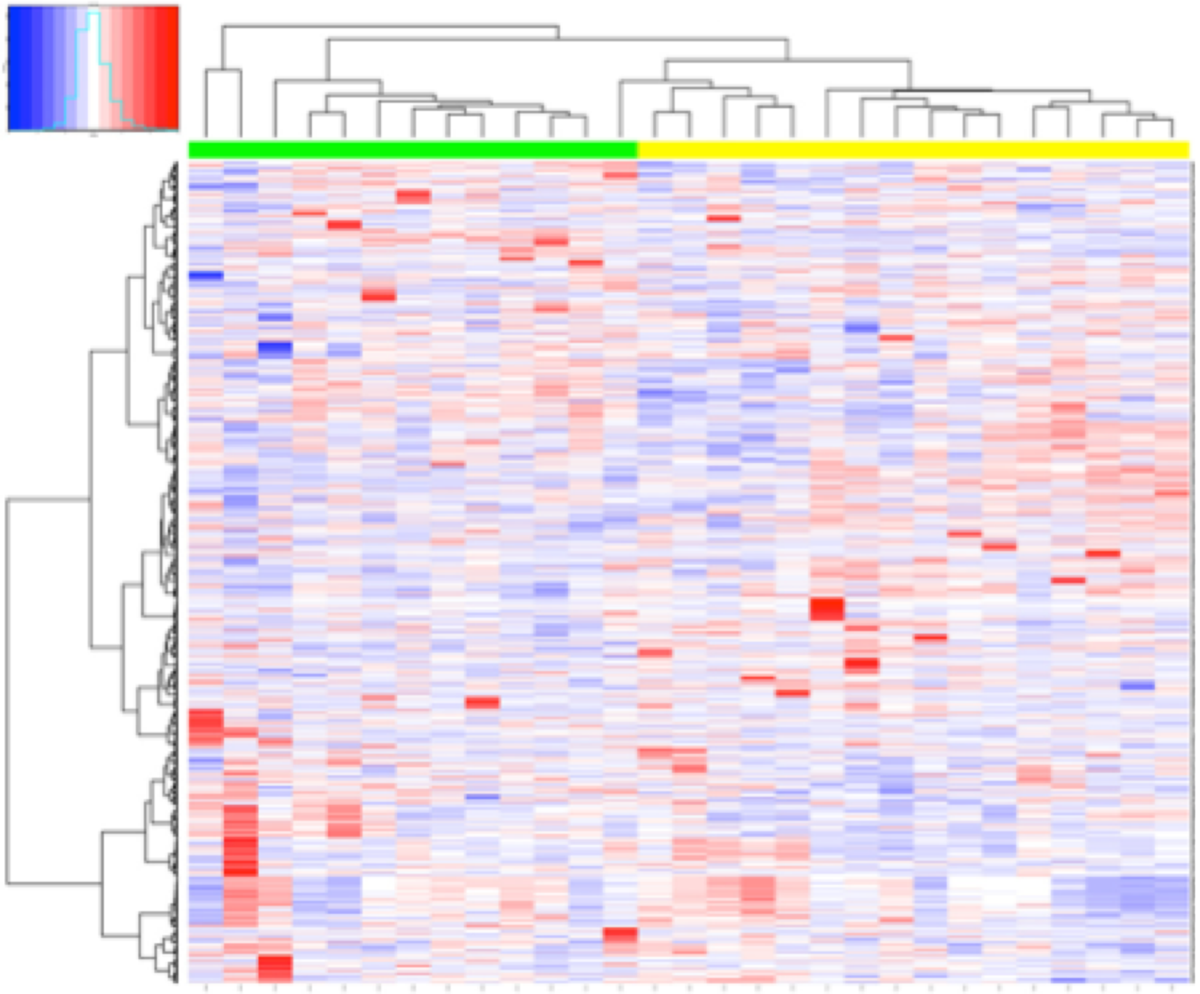

Additional Figure 1: Unfiltered clustering diagram of all 1129 measured proteins

Unsupervised hierarchal clustering of lcSSc-PAH patients (green color bar) and lcSSc patients with no lung disease (yellow color bar). Here, red and blue indicate high or low expression
