# Supplementary material for: Serum biomarker for diagnostic evaluation of pulmonary arterial hypertension in systemic sclerosis
Source: Arthritis Res Ther. 2018 Aug 16;20:185. doi: 10.1186/s13075-018-1679-8 (PMC6097341; doi:10.1186/s13075-018-1679-8)
Supplement: Supplementary file 5 — Figure S2. Receiver operator characteristic (ROC) curves of SOMAlogic: ROCs based on in silico (logistic regression). (PDF 678 kb) [file 13075_2018_1679_MOESM5_ESM.pdf]

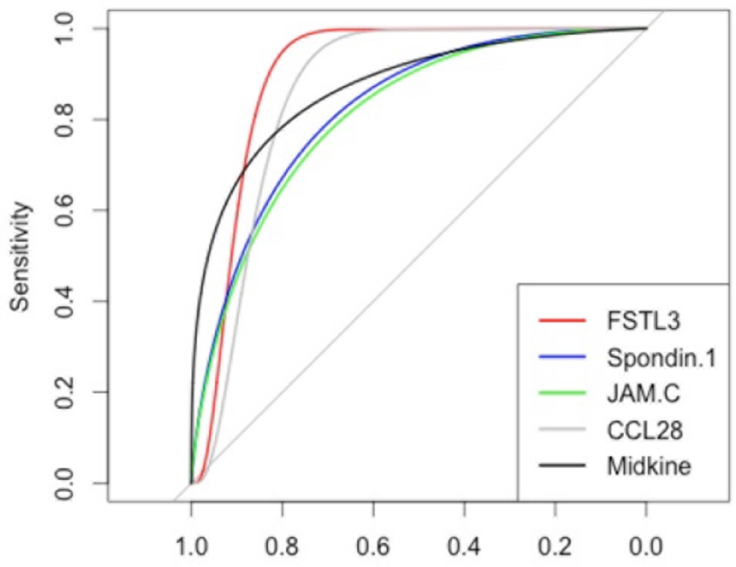

Protein	AUC	CI	Sensitivity	Specificity
FSTL3	0.90	0.75-0.95	0.96	0.79
Spondin-1	0.84	0.65-0.93	0.78	0.71
JAM-C	0.82	0.62-0.92	0.77	0.70
CCL28	0.87	0.69-0.94	0.94	0.72
Midkine	0.89	0.72-0.95	0.76	0.82

Additional Figure 2: Receiver operator characteristic curves of SOMAlogic
Receiver operator characteristic curves (ROCs) based on in silico (logistic regression)
