# Supplementary material for: Serum biomarker for diagnostic evaluation of pulmonary arterial hypertension in systemic sclerosis
Source: Arthritis Res Ther. 2018 Aug 16;20:185. doi: 10.1186/s13075-018-1679-8 (PMC6097341; doi:10.1186/s13075-018-1679-8)
Supplement: Supplementary file 6 — Figure S3. Correlations of Midkine (MDK), follistatin-like 3 (FSTL3), and BNP: protein levels of MDK, FSTL3, and BNP are plotted as linear regressions Spearman’s correlation coefficients are reported in the upper left corner of each graphic. (A) Examines the correlation between FSTL3 and MDK, (B) the correlation between FSTL3 and BNP, (C) MDK and BNP. (PDF 361 kb) [file 13075_2018_1679_MOESM6_ESM.pdf]

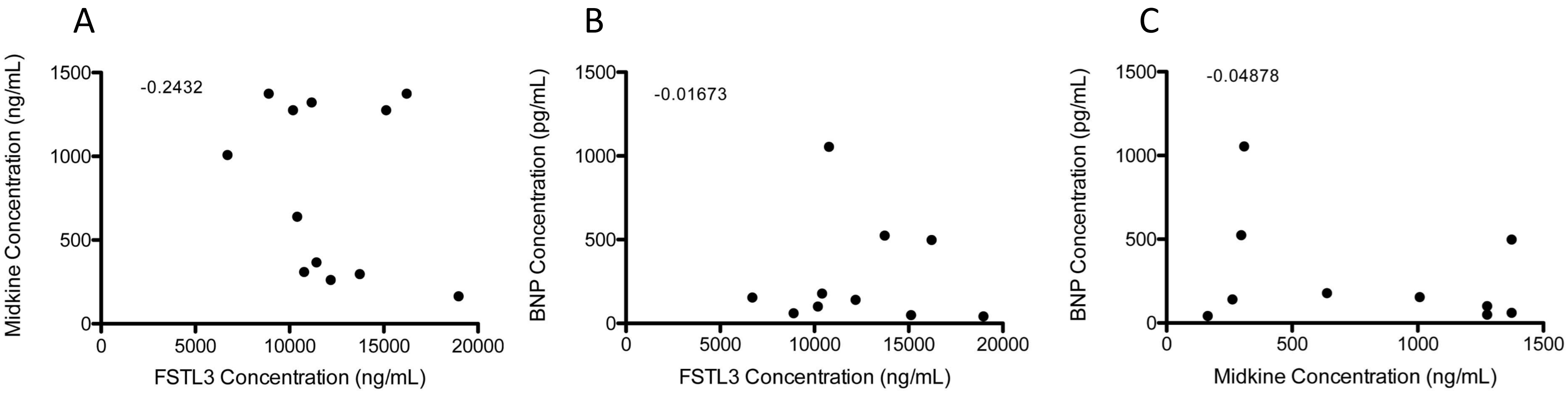

Additional Figure 3: Correlations of Midkine, FSTL3 and BNP

Protein levels of Midkine, FSTL3 and BNP are plotted as linear regressions spearman's correlation coefficients are reported in the upper left corner of each graphic. A examines the correlation between FSTL3 and Midkine, B the correlation between FSTL3 and BNP, C Midkine and BNP
