# Supplementary material for: Serum biomarker for diagnostic evaluation of pulmonary arterial hypertension in systemic sclerosis
Source: Arthritis Res Ther. 2018 Aug 16;20:185. doi: 10.1186/s13075-018-1679-8 (PMC6097341; doi:10.1186/s13075-018-1679-8)
Supplement: Supplementary file 7 — Figure S4. Concentrations of Midkine (MDK), follistatin-like 3 (FSTL3): concentrations of FSTL3 (A) and Midkine (B) were measured by ELISA and modeled together using multiple logistic regression (C). (PDF 779 kb) [file 13075_2018_1679_MOESM7_ESM.pdf]

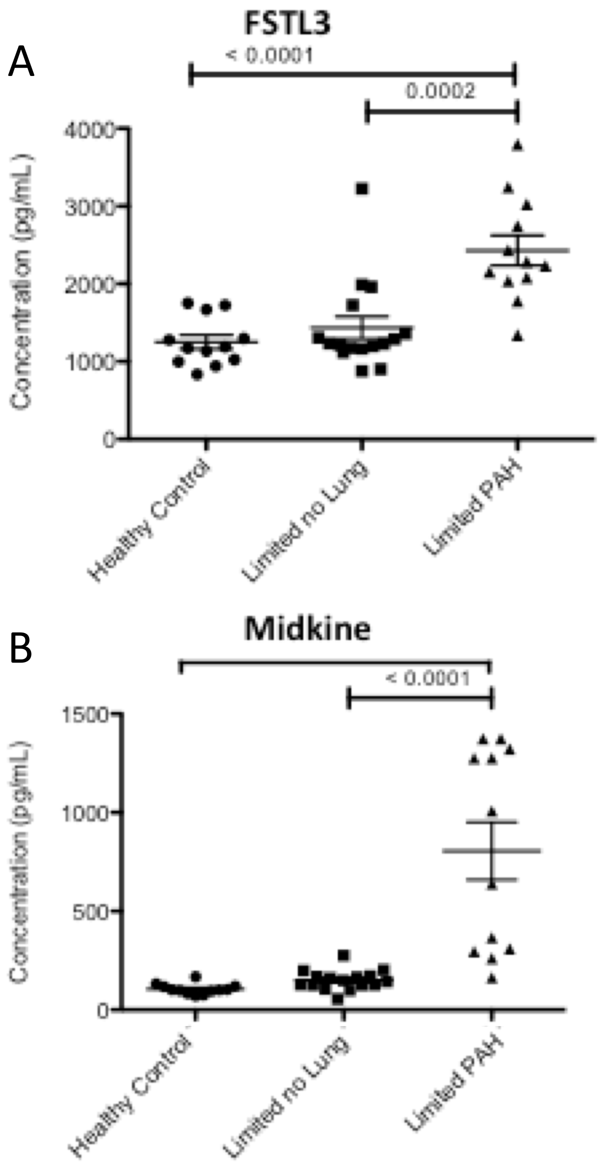

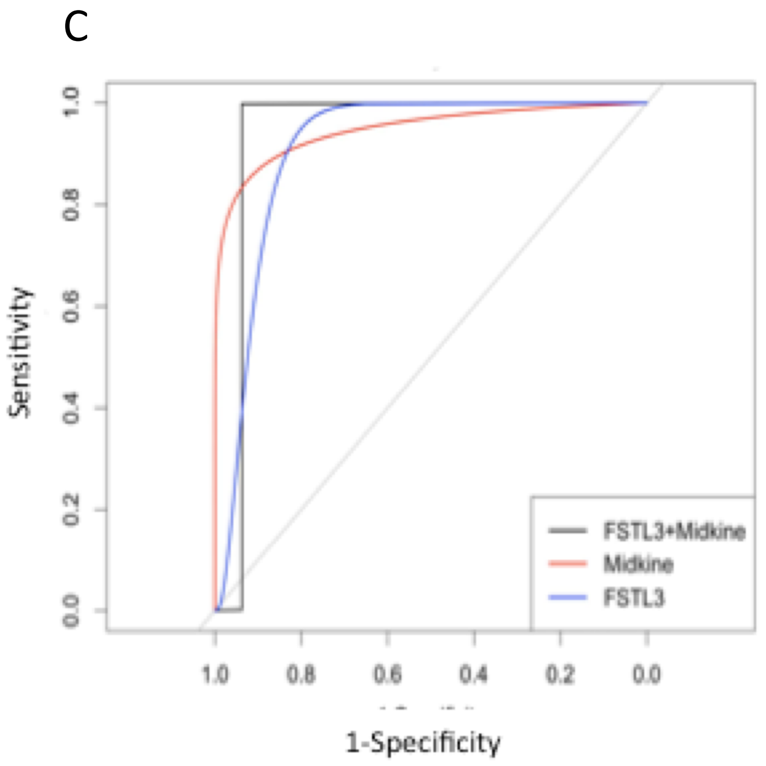

Protein	AUC	CI	Sensitivity	Specificity
FSTL3	0.91	0.75-0.95	0.95	0.80
Midkine	0.94	0.80-0.96	0.84	0.93
FSTL3+Midkine	0.99	0.98-1.0	0.99	0.94

Additional Figure 4: Concentrations of Midkine, FSTL3

Concentrations of FSTL3 (A) and Midkine (B) were measured by ELISA and modeled together using multiple logistic regression (C)
