# Supplementary material for: Serum biomarker for diagnostic evaluation of pulmonary arterial hypertension in systemic sclerosis
Source: Arthritis Res Ther. 2018 Aug 16;20:185. doi: 10.1186/s13075-018-1679-8 (PMC6097341; doi:10.1186/s13075-018-1679-8)
Supplement: Supplementary file 8 — Table S4. Detailed hemodynamic measurements and clinical characteristics of validation cohorts: Table A contains the details from validation cohort one while Table B contains the details from validation cohort two. (PDF 334 kb) [file 13075_2018_1679_MOESM8_ESM.pdf]

A

								Hemodynamic Measurements							
StudyID	Sex	Age	PAH	Treated	Treatment	Sample at RHC	mPCWP (mmHg)	mPAP (mmHg)	PVR (Woods Units)	BNP w/in 1 yr of RHC	BNP (pg/mL)	ILD	HRCT	w/in 1 yr of	PASP (mmHg)
Tufts_403	F	54	YES	YES	PDE-5	YES	15	47	6.4	YES	157	NO	-	-	-
Tufts_333	F	77	YES	No	-	YES	5	60	27.0	YES	926	NO	-	-	-
Tufts_347	M	80	YES	No	-	YES	11	43	6.4	YES	962	NO	-	-	-
Tufts_351	F	61	YES	No	-	YES	7	51	7.8	YES	64	NO	-	-	-
Tufts_003	F	82	YES	No	-	YES	15	53	7.9	No	1630	YES	-	-	-
Tufts_014	F	70	YES	YES	Ca Channel Blk	YES	13	37	7.5	YES	78	NO	-	-	-
Tufts_101	F	85	YES	No	-	YES	8	27	4.2	YES	64	NO	-	-	-
Tufts_106	F	67	YES	YES	PGE-2	YES	13	34	4.0	YES	64	NO	-	-	-
Tufts_265	F	58	YES	No	ERA and PDE-5	YES	7	30	3.5	YES	138	NO	-	-	-
Tufts_278	M	61	YES	No	-	YES	10	34	3.9	YES	12	NO	-	-	-
Tufts_395	M	68	YES	No	-	YES	13	46	7.3	YES	129	NO	-	-	-
1323	F	60	YES	No		YES	-	69	10	-	-	NO			
1405	F	67	YES	YES	ACE and Hydroxychloroquine	YES	14	26	3.00	YES	127	NO			
1481	F	71	YES	No	ACE	YES	4	48	11.0	YES	248	NO			
1483	F	63	YES	YES	Hydroxychloroquine	YES	10	57	11.6	YES	234	YES			
1815	F	64	YES	YES	PGE-2	YES	1	47	13.4	YES	1408	NO			
1176	F	66	YES	YES	Corticosteroid and ACE	YES*	14	32	3.4	YES	411	NO			
1507	F	62	YES	YES	PGE-2	**YES**	3	41	**11.2**	YES	1277	NO			
1492	F	58	YES	YES	Cyclo	NO (22 days after)	14	46	6.2	YES	414	YES			
1779	F	64	YES	YES	ACE	YES	8	60	12.3	YES	465	NO			
1477	F	73	YES	No		NO (1 month before)	15	29	3.3	YES	110	NO			
1716	M	52	YES	YES	Corticosteroid and ACE	YES	7	31	3.9	YES	42	NO			
1436	F	59	YES	No		YES	12	26	3.8	YES	89	NO			

*blood taken within a week

B

							Hemodynamic Measurements					
StudyID	Sex	Age	PAH	Treated	Treatment	Sample at RHC	mPCWP (mmHg)	mPAP (mmHg)	PVR (Woods Units)	BNP w/in 1 yr of RHC	BNP (pg/mL)	ILD
P1	**F**	70	YES	YES	ARB, Ca Channel Blk	YES	10	54	12.90	No	-	NO
P2	**F**	56	YES	YES	Ca Channel Blk	No (3 months before)	15	42	4.96	Yes	88	NO
P3	**F**	73	YES	YES	ACE	No (3 years before)	15	39	missing	No	-	NO
P7	**F**	66	YES	YES	Ca Channel Blk	No (2 years before)	7	28	3.23	No	-	NO
P8	**F**	70	YES	YES	ACE	YES*	8	49	missing	Yes	1333	NO
P10	**F**	81	YES	YES	Ca Channel Blk	YES	9	26	missing	Yes	84	NO
P11	**F**	56	YES	YES	ACE	No (1 year before)	10	51	7.24	No	-	NO
P12	**F**	66	YES	YES	ACE	No (3 months after)	5	32	5.91	No	-	NO
P13	**F**	52	YES	YES	Ca Channel Blk	No (3 months before)	8	44	missing	Yes	449	NO
P14	**F**	57	YES	YES	ACE, PGE-2, Immunosupressent	No (2 years before)	missing	25	missing	No	-	NO
P16	**F**	74	YES	YES	ACE, Ca Channel Blk	YES	8	51	12.65	No	-	NO

Additional Table 4: Detailed hemodynamic measurements and clinical characteristics of validation cohorts

Table A contains the details from validation cohort one while Table B contains the details from validation cohort two
