# Supplementary material for: Serum biomarker for diagnostic evaluation of pulmonary arterial hypertension in systemic sclerosis
Source: Arthritis Res Ther. 2018 Aug 16;20:185. doi: 10.1186/s13075-018-1679-8 (PMC6097341; doi:10.1186/s13075-018-1679-8)
Supplement: Supplementary file 9 — Figure S5. Concentrations of Midkine (MDK), follistatin-like 3 (FSTL3): concentrations of FSTL3 and MDK were examined with treated subjects excluded from the analysis (A) or subjects with ILD (B). ROC parameters were examined with the exclusion of the different subsets (C). (PDF 970 kb) [file 13075_2018_1679_MOESM9_ESM.pdf]

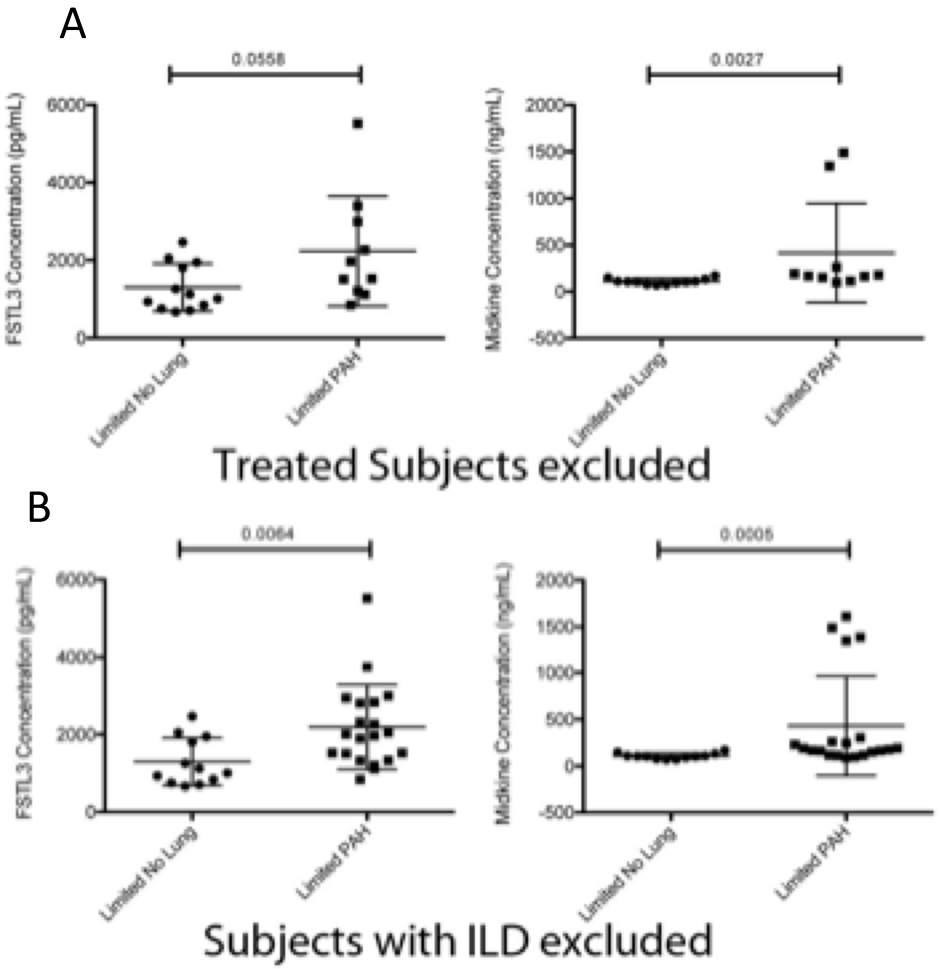

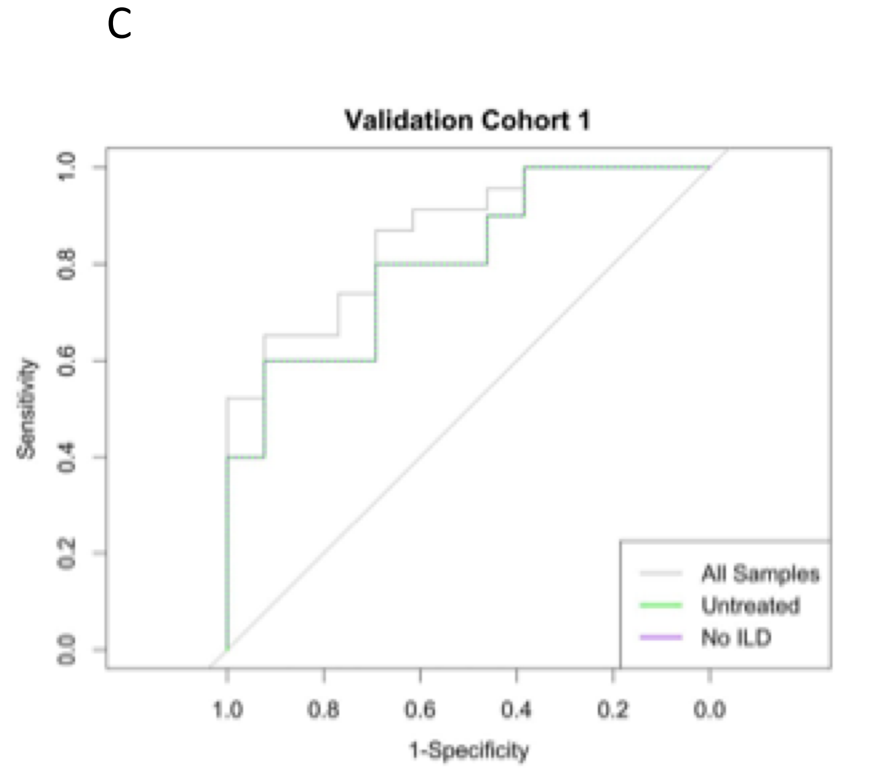

	Protein	AUC	CI	Sensitivity	Specificity
All Subjects	FSTL3 Midkine	0.85	0.67-0.94	0.76	0.76
Treated Subjects Excluded	FSTL3 Midkine	0.79	0.55-0.90	0.67	0.76
Subjects with ILD Excluded	FSTL3 Midkine	0.82	0.62-0.93	0.76	0.73

Additional Figure 5: Concentrations of Midkine, FSTL3

Concentrations of FSTL3 and Midkine were examined with treated subjects excluded from the analysis (A) or subjects with ILD (B). ROC parameters were examined with the exclusion of the different subsets (C)
