# Supplementary material for: Serum biomarker for diagnostic evaluation of pulmonary arterial hypertension in systemic sclerosis
Source: Arthritis Res Ther. 2018 Aug 16;20:185. doi: 10.1186/s13075-018-1679-8 (PMC6097341; doi:10.1186/s13075-018-1679-8)
Supplement: Supplementary file 4 — Table S3. Shortlist proteins previously observed as differentially regulated in skin and lung tissue or PBMCs of SSc-PAH patients. (PDF 71 kb) [file 13075_2018_1679_MOESM4_ESM.pdf]

Additional Table 3: Shortlist proteins previously observed as differentially regulated in skin and lung tissue or PBMCs of SSc-PAH patients

	Somascan			PBMCs (Lafyatis)			PBMCs (Bull)			PBMCs (Hassoun)			Lung Tissue (Feghali-Bostwick)			Skin Tissue (Assassi)		
**Gene Symbol**	*Log FC*	*P.value*	*Adj P.value*	*Log FC*	*P.value*	*Adj P.value*	*Log FC*	*P.value*	*Adj P.value*	*Fold Change*	*Geo Mean SSc*	*Geo Mean HC*	*Log FC*	*P.value*	*Adj P.value*	*Fold Change*	*Geo Mean SSc*	*Geo Mean HC*
**MDK**	2.54	0.010	0.15	-	-	-	-	-	-	-	-	-	1.41	0.000	0.03	1.65	346	210
**SFRP1**	2.50	0.009	0.10	-	-	-	-	-	-	-	-	-	-	-	-	1.70	868	510
**TNC**	0.65	0.010	0.10	-	-	-	-	-	-	-	-	-	-	-	-	1.85	493	266
**GFRA1**	0.64	0.004	0.08	-	-	-	2.38	0.003	0.09	-	-	-	-	-	-	-	-	-
**ANGPTL4**	0.64	0.004	0.08	-	-	-	-	-	-	-	-	-	-	-	-	1.31	371	284
**SPON1**	0.55	0.002	0.06	-	-	-	-	-	-	-	-	-	-	-	-	1.45	430	298
**FSTL3**	0.55	0.000	0.04	-	-	-	-	-	-	-	-	-	-	-	-	1.26	138	110
**RARRES2**	0.54	0.000	0.03	-	-	-	-	-	-	-	-	-	-	-	-	1.37	353	258
**CCL19**	0.50	0.003	0.08	-	-	-				-	-	-	-	-	-	1.76	538	306
**TIMP1**	0.41	0.001	0.04	0.409	0.002	0.15	0.69	0.000	0.03	-	-	-	-	-	-	1.51	3215	2125
**CD27**	-0.23	0.010	0.15	-	-	-	-0.77	0.000	0.04	-	-	-	-	-	-	1.17	56	48
**LGALS3**	-0.29	0.009	0.15	-	-	-	0.64	0.001	0.05	-	-	-	-	-	-	-	-	-
**CD55**	-0.37	0.002	0.07	-	-	-	0.51	0.001	0.07	-	-	-	-	-	-	-	-	-
**CD36**	-0.49	0.008	0.14	-	-	-	0.48	0.002	0.08	-	-	-	-	-	-	-	-	-
